# Inkjet printing of blue phosphorescent light-emitting layer based on bis(3,5-di(9*H*-carbazol-9-yl))diphenylsilane

**DOI:** 10.1039/c8ra00582f

**Published:** 2018-03-20

**Authors:** Robert Bail, Ji Yoon Hong, Byung Doo Chin

**Affiliations:** Department of Convergent Systems Engineering, Dankook University Yongin 16890 South Korea; Department of Polymer Science and Engineering, Dankook University Yongin 16890 South Korea bdchin@dankook.ac.kr

## Abstract

In this study, micropatterning of a blue light emitting, tetraphenylsilane-based phosphorescent material by inkjet printing was investigated. Bis(3,5-di(9*H*-carbazol-9-yl))diphenylsilane (SimCP2) doped with iridium bis(4,6-difluorophenypyridinato)picolate (FIrpic) was dissolved in a solvent mixture, and various conditions for the solvent composition and drying of films were examined. Homogeneous dot and line patterns with controllable thickness and smooth surface were obtained from a mixture of chlorobenzene and cyclohexanone at a moderate printing speed of 3 mm s^−1^ and a droplet ejection frequency of 70 Hz. An inkjet-printed device was designed and fabricated in [ITO/PEDOT:PSS /PVK/SimCP2:Flrpic/TSPO1/TPBi/LiF/Al] configuration, from which sky-blue light (0.14, 0.25) was obtained with a luminous efficiency of 10.73 cd A^−1^ and a power efficiency of 6.13 lm W^−1^. This amounted to 68% of the performance of an identical device where the emitting layer was spin coated. These results show the potential of inkjet printing as a low-cost patterning method for low molecular weight emitters in blue light emitting devices.

## Abstract 2

This study demonstrates successful inkjet printing of SimCP2 doped with FIrpic for applications in large area blue phosphorescent OLEDs.

## Graphical abstract

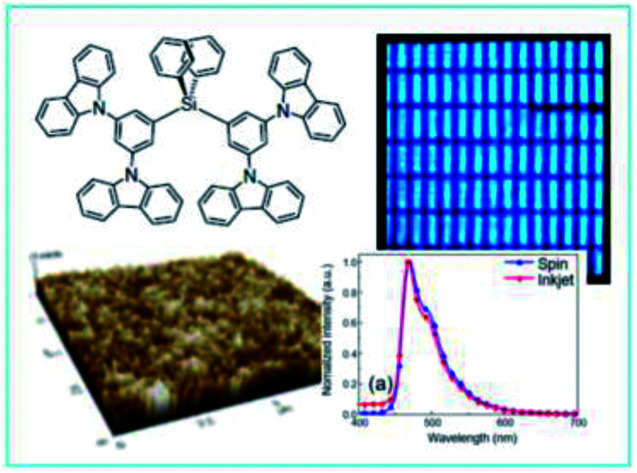

## Introduction

There is a need for more efficient and stable molecular materials for blue phosphorescent organic light-emitting diodes (OLEDs).^1^ Hosts of blue-phosphorescent emitters generally require well-balanced transport properties for both holes and electrons, and superior triplet energy compared with that of the dopant to suppress the detrimental triplet–triplet annihilation phenomenon.^2^ Among others, carbazole-based functional compounds have been identified as suitable small molecular hosts for triplet-state blue-emitting dopants such as iridium bis(4,6-difluorophenypyridinato)picolate (Flrpic). The feasibility of incorporating Flrpic in 1,3-bis(9-carbazolyl)benzene (mCP) was demonstrated by Holmes *et al.*, yielding an external quantum efficiency (*η*_ext_) of around 7.5% and a luminous power efficiency of 8.0–9.8 lm W^−1^.^3^ The performance can be further increased if one or several mCP unit(s) are incorporated in tetraphenylsilane, as demonstrated for 3,5-bis(9-carbazolyl) tetraphenylsilane (SimCP) and bis(3,5-di(9*H*-carbazol-9-yl))diphenylsilane (SimCP2), for example. Devices with these host materials and doped with Flrpic showed enhanced efficiencies, with *η*_ext_ = 14.4% and 12.9 lm W^−1^ reported for SimCP,^4,5^ and *η*_ext_ = 17.7% and 24.2 lm W^−1^ stated for SimCP2.^6,7^ It has also been demonstrated that thin layers of these carbazole-based hosts can be deposited not only by vacuum techniques but also by solution processing (spin coating), resulting in extraordinarily smooth surface morphologies and quantum efficiencies of up to 21.0%.^8^ These results demonstrate the potential of this small molecular host for applications in solution-processed OLEDs.

Among the fabrication methods for solution-processed OLEDs, drop-on-demand (DOD) piezo inkjet printing has attracted some research interest as a potential alternative to vacuum processing for large area OLEDs.^9^ It has been demonstrated that green- and red-emitting OLEDs with relatively high quantum and power efficiencies (11.7%, 29.9 lm W^−1^) can be conveniently fabricated by inkjet printing from small molecular host materials commonly used in vacuum processes like 4,4′-bis(carbazol-9-yl)biphenyl (CBP), for instance.^10^ The literature is less extensive with regard to inkjet-printed blue emitting materials but an example was given by Xing *et al.* who deposited uniform thin films from an ink containing poly(9,9-di-*n*-octylfluorene) (PFO) as a host with sufficiently high triplet-state energy.^11^ In general, inkjet-printed OLED devices benefit from the characteristics of small molecular host materials including their purity, chemical stability and high triplet energy. However, solution processing this type of material can lead to uneven thickness, rough surfaces and morphological instabilities of the emitting layer, which is detrimental for the efficiency and lifetime of a device.^12^ Moreover, hosts for blue phosphorescent emitters are limited to those with sufficiently high triplet energy. In other words, careful ink formulation and process conditioning is required for uniform printing of small molecular host materials.

The so-called “coffee-ring” effect is a common phenomenon with inkjet printing, as demonstrated with an ink containing dithienylbenzothiadiazole dissolved in a solvent mixture of *p*-xylene and 3,4-dimethylanisole.^13^ This term refers to ring-shaped stains of solids that accumulate along the perimeter of a drop after evaporation of the liquid phase.^14^ The formation of these characteristic patterns can be ascribed to a replenishing capillary flow inside a drying drop that always occurs if the solvent rapidly evaporates, the contact line is pinned to its initial position, and the contact angle is larger than 0°. Well-defined structures with concave or even plateau-like cross-sections can be accomplished when the internal solute flow inside a drying droplet is restrained with respect to the evaporation dynamics. This can be achieved in several ways, like combining a low- with a high-boiling point solvent,^15^ reversing the outward capillary movement *via* Marangoni flow,^16^ controlling droplet spacing^17^ or droplet size,^18^ or increasing the ink viscosity.^19^ Unpinning the contact line by introducing a perfluorinated layer also significantly improves print results.^20^

SimCP2 is a small-molecular host that enables comparatively high efficiency in blue phosphorescent organic light-emitting diodes, and it can be deposited either by vacuum processing or by solution processing.^7,8^ However, there is a lack of information on how this type of material can be selectively deposited by inkjet printing, which is potentially attractive for the fabrication of large area OLED displays. Our aim was to demonstrate that homogeneous thin-film patterns made of SimCP2 doped with Flrpic can be formed by inkjet printing and proper ink formulation. For this purpose, a solvent combination with good host compatibility was identified. Subsequently, the effect of the solvent ratio in the ink on the characteristics of the deposited host patterns was investigated, and the effect of the print speed on thickness, morphology and surface roughness of the produced films was assessed. Finally, a test device was made and its performance was measured. The insights gained will be useful in the future development and employment of functional ink formulations for inkjet-printed blue OLEDs.

## Experimental

### Material preparations

Indium-tin oxide (ITO, 100 nm) coated glass sheets were deeply cleaned (sonicated in IPA : Acetone (50 : 50) for 15 min → sonicated in chloroform for 10 min → sonicated in IPA (20 °C) for 15 min → boiled (250 °C) in IPA for 10 min), and then air-dried. To create a hole injection layer (HIL), the raw substrates were spin coated (3000 rpm, 30 s) with PEDOT:PSS dissolved in IPA (50/50, v/v) and annealed at 150 °C for 10 min (hotplate). A buffer layer was added by spin coating (2000 rpm, 30 s) the substrates with a poly(9-vinylcarbazole) (PVK) solution (0.2 wt%, in chlorobenzene), followed by reheating (100 °C, 20 min) to remove residual moisture prior to deposition of the ink. PEDOT:PSS (AI4083, Heraeus Korea) and PVK (*M*_w_ ∼ 1 100 000, Sigma Aldrich Korea) were used with no further purification.

Iridium bis(4,6-difluorophenypyridinato)picolate (Flrpic) was added to SimCP2 at a ratio of 1 : 10 (w/w). The dopant–host mixture was dissolved in each of six flasks containing 1.2 mL of a two-component solvent. The latter consisted of chlorobenzene (CB) as the low-boiling solvent and cyclohexanone (CHX) as the cosolvent. The properties of the solvents used in this study are given in Table 1. The solvent ratio was altered from 100 : 0 to 50 : 50 in steps of 10 mol%. FIrpic (OSM Korea Ltd) and SimCP2 (LumTec, Taiwan) were used as received.

**Table 1:** Properties of solvents used in this study^21^

Solvents	Parameters		
	Boiling point (°C)/vapour pressure (mmHg)	Viscosity (cP)	Surface tension (mN m^−1^)
Chlorobenzene (CB)	131.7/11.2	0.806	33.1
Cyclohexanone (CHX)	155.7/3.0	2.02	32.5

### Ink deposits and characterization

An R&D inkjet printing system (OmniJet 300, Unijet, Seongnam, Korea) with a Dimatix print head (DMC-11610, 21 μm nozzle diameter) served as the patterning unit. Throughout all experiments, the print bed temperature was set to 30 °C. Identical dot arrays were produced at a drop ejection frequency of 50 Hz for each ink (0 to 50 mol% cosolvent). The patterned substrates were removed from the printer bed and heated for 30 min to evaporate all solvent residues (hotplate, 120 °C). Following characterization of the dried host patterns, the best among the tested cosolvent concentrations was identified. Using the latter, line patterns were obtained at a constant drop pitch of 50 μm for a range of drop ejection frequencies and print head velocities (50 Hz at 1 mm s^−1^ to 110 Hz at 7 mm s^−1^).

The patterned samples were placed under the CCD zoom camera (×5) of a dark-shielded probe station (MST-8000C, MS TECH, Hwaseong, Korea). With the samples exposed to UV light (254 nm) emitted by a handheld device, images of the luminescent patterns were taken in image analysis software (i-SolutionTM, IMT Inc., Daejeon, Korea). Subsequently, cross-sectional profiles of the printed dots/lines were generated on a surface profiler (Veeco Dektak 150) with a stylus tip diameter of 12.5 μm at a scanning speed of 10 μm s^−1^.

### OLED test devices

**Fig. 1:**
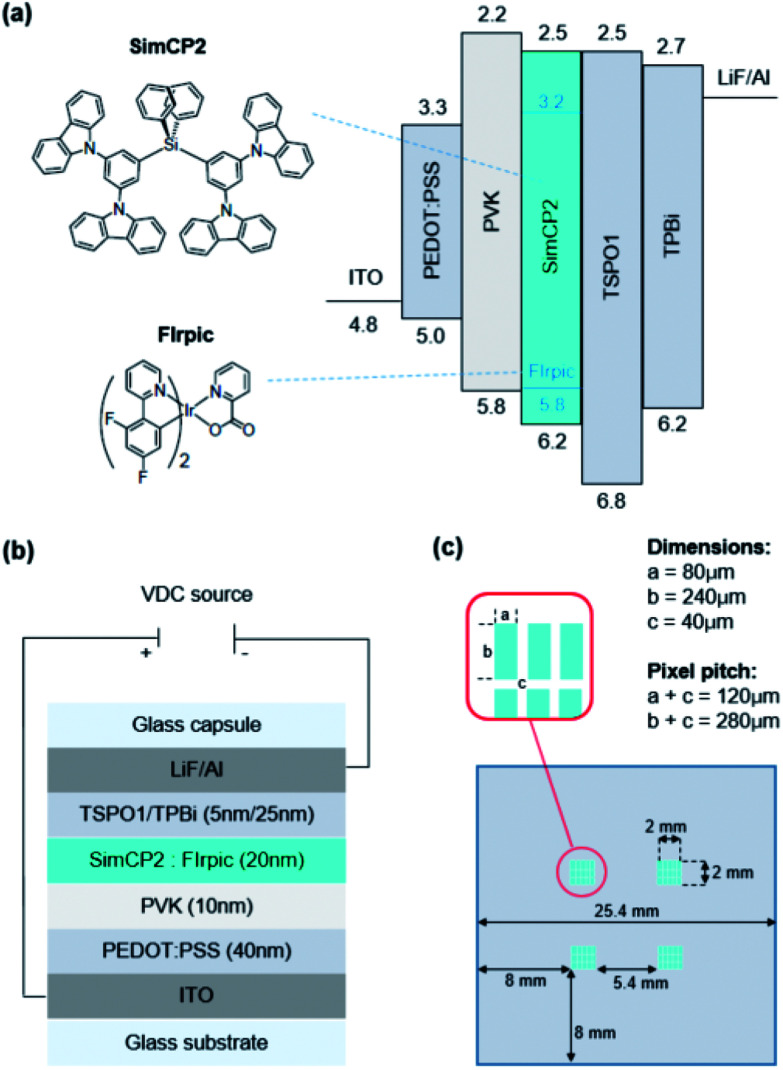
Test device architecture showing (a) energy gap levels of layered materials, (b) device configuration, and (c) pixel-pattern of OLED test device.

To validate the findings from this study and to illustrate the effects of the ink and the inkjet printing process on the device characteristics, an OLED device was designed as outlined in Fig. 1.

The devices were fabricated in two different versions, which merely differed in terms of how the emitting layer (EML) was deposited. For both, an ITO-coated glass sheet was cut to size and cleaned as previously described. A pixel-patterned well structure was created by spin coating (60 s, 1000 rpm) a 500 nm thin layer of a photoresist (SU-8) onto each chip, before exposing it to a patterned mask illuminated with UV light (254 nm, 20 s). The pixel dimensions were 80 μm and the sup-pixel pitch was 120 μm, which corresponded to approximately 70 ppi. More details regarding the layout of the pixel-patterned cell are given in Fig. 1(c).

The pre-patterned substrates were coated with PEDOT:PSS and PVK as previously described. The EML was then deposited from the same ink (5.5 mg mL^−1^ FIrpic/SimCP2 in 1.2 mL CB:CHX) by inkjet printing (Omnijet 300, optimum print speed) and by spin coating (3000 rpm, 30 s) on the main device and the reference device, respectively. Following annealing (120 °C, 30 min), each device was coated with two electron-transport layers (TSPO1/TPBi) and two cathode layers (LiF/Al) by thermal vapor deposition (DOV, Korea) at growth rates of 1 Å s^−1^ (TSPO1, TPBi, Al) and 0.1 Å s^−1^ (LiF), respectively.

The surface characteristics of the spin-coated and inkjet-printed dried layers were visualized by atomic force microscopy (Veeco MMAFM-2/1441EX), which also measured the surface roughness (RMS) according to ASME B46.1-2009. The spectral output and efficiency data for each device were obtained by analysis using a Keithley 2400 source unit paired with an OLED *I*–*V*–*L* measuring system (PR-655 SpectraScan, Photo Research, Inc., USA). Commission internationale de l'éclairage (CIE 1931) parameters were obtained from the integrated spectrophotometer. The current density (*J*) is defined in eqn (1)

1 *J* = *I*/*A*,

where *I* is the electric current in milliamps [mA], and *A* is the cross-sectional area [cm^2^]. The actual pixel surface was considered in the calculation in the form of the actual-to-nominal pixel surface (aperture ratio, in percent). The current efficiency (*E*) of the OLED devices was derived as outlined in eqn (2)

2 *E* = *I*(*V* )/*I*,

where *I*(*V*) is the luminous intensity measured in candela [cd], and *I* is the electric current in amps [A]. The power efficiency (PE) was calculated according to eqn (3)

3 PE = *Φ*(*V *)/*P*,

where *Φ*(*V*) is the luminous flux in lumen [lm], and *P* is the electric power in watt [W].

## Results and discussion

### Effect of solvent ratio

**Fig. 2:**
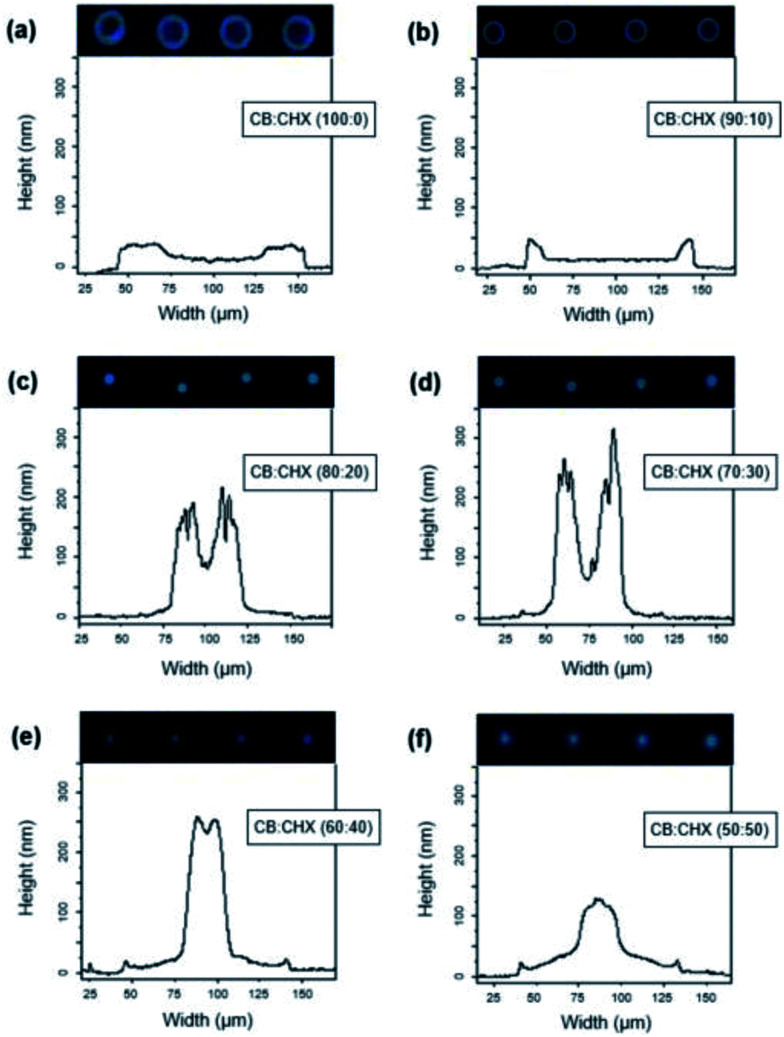
Effect of the CB : CHX solvent ratio on printed-dot morphologies.

In an initial test print, dot patterns were produced from an ink containing the FIrpic-doped host dissolved in pure chlorobenzene (CB : CHX = 100 : 0). The dry dot patterns measured 130 μm in diameter, the largest in comparison. The layer thickness amounted to 10–18 nm at the dot center and 30–40 nm along the thick edges, indicating coffee staining. The image of the dot morphology shown in Fig. 2(a) suggested a certain level of host aggregation. The pitted character of the related surface profile, indicating a rough surface, further substantiated this. To improve the film properties, CHX was added in the ink formulation as a viscous cosolvent with a moderately higher boiling point and hence lower vapor pressure compared to CB.

The follow-up experiments (dot arrays) revealed strong influence of the cosolvent concentration on the shape of the dried dots. Addition of only 10% CHX considerably improved the thin film properties as shown in Fig. 2(b). Perfectly circular dots with continuous edges were produced. The dot diameter amounted to 90–100 μm and the film thickness at the dot center slightly increased to 15–20 nm. A much less serrated cross-sectional profile pointed at a relatively smooth surface. Moderate coffee rings remained though, indicating that the cosolvent concentration may have been slightly too low.

With a further increase in the cosolvent concentration, the shape and morphology of the dried dot patterns dramatically changed as shown in Fig. 2(c) and (d) for the CB : CHX-solvent ratios of 80 : 20 and 70 : 30, respectively. The dot diameter halved to around 45–50 μm, and the material piled up in the vertical direction. At 20% CHX, the film thickness reached up to 80 nm at the dot center and a maximum of 200 nm along the dot edges. The latter were even taller (250–300 nm) at a CHX content of 30%. Another change in dot shape and morphology was revealed for the two lowest CB : CHX solvent ratios of 60 : 40 and 50 : 50 as displayed in Fig. 2(e) and (f), respectively. The majority of the host material accumulated within a zone of approximately 30 μm in diameter at the center of the dots. This led to about 250 nm (40% CHX) and 130 nm (50% CHX) tall deposits. However, deposits that concentrated in the central dot area replaced the previously observed holed dot centers.

The observations made in the dot printing experiments reflect the impact of the two utilized solvents and their ratio on the ink properties, which in turn determine the evaporation dynamics in a drying droplet. Used as the main solvent, chlorobenzene has a relatively low boiling point (high vapor pressure) and low viscosity (0.81 cP). The surface tension is not particularly high or low. Cyclohexanone was chosen as the cosolvent because it has a 24 °C higher boiling point and a remarkably high viscosity (2.02 cP), while the surface tension is in a similar range.^21^ Therefore, the observed dot morphologies can be mainly interpreted as the result of overlapping vapor-pressure and viscosity effects.

The initial ink formulation (doped host in 100% CB) evaporated fast and had low viscosity, similar to that of pure chlorobenzene. Due to these characteristics, the capillary solvent flow inside the drying droplet was not suppressed, which in turn resulted in characteristic coffee-ring deposits und rough surface morphologies as seen in Fig. 2(a). Addition of 10% CHX slightly lowered the vapor pressure of the ink and marginally extended the time span required to evaporate all solvent. More importantly, the ink viscosity increased at the same time, which hindered solute particle movement during drying to some extent. This would explain why the deposits showed a smoother surface and less pronounced coffee rings in Fig. 2(b).

By contrast, elevated CHX concentrations of 40–50% considerably increased ink viscosity, which induced a dominant surface tension gradient leading to Marangoni flow. The latter channeled the majority of the host molecules to the droplet center, which was obvious in Fig. 2(e) and in particular in Fig. 2(f).

At intermediate solvent ratios (80 : 20 and 70 : 30), a combination of the two flow types may have taken place. Our hypothesis is that Marangoni flow (surface tension gradient) may have dominated the beginning of the drying process, piling up large amounts of the host material at the center of the droplet. Once the chlorobenzene had evaporated, the surface tension gradient weakened and capillary flow was established somewhat later in the drying process. This would explain why the profiles in Fig. 2(c) and (d) exhibited top surfaces with steep coffee rings and a deeply hollowed-out center.

In consideration of these results, the solvent ratio was adjusted to 85 : 15 (CB : CHX) for all follow-up trials and the test device. This aimed at increasing the film thickness at the center of the pattern while further reducing the coffee-ring effect.

### Effect of ejection frequency

**Fig. 3:**
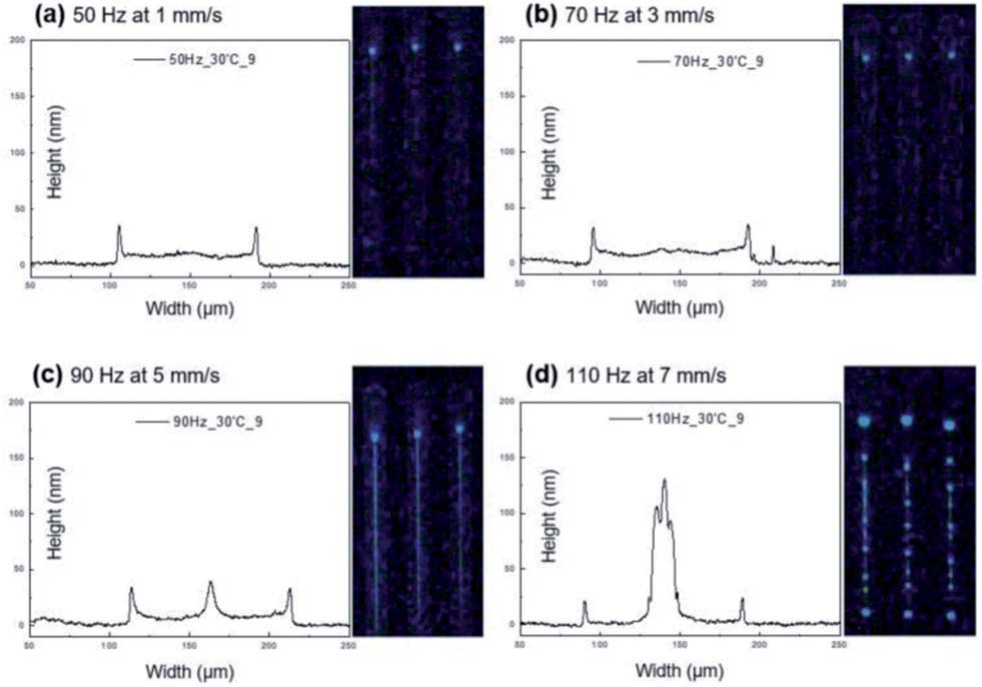
Effect of ejection frequency (50–110 Hz) on printed line morphologies.

A series of line patterning experiments was conducted over a range of four different drop ejection frequency settings (50, 70, 90, and 110 Hz). Evenly deposited, continuous line patterns were obtained with the refined ink formulation when printed at 50 and 70 Hz as indicated by Fig. 3(a) and (b). The lines were approximately 15–20 nm high in the center area and 90 μm wide. The line features exhibited smooth surfaces but minor coffee staining still persisted as indicated by circa 35 nm high walls on both sides of the cross sectional profiles.

The line pattern characteristics changed at a frequency of 90 Hz as seen in Fig. 3(c). A circa 45 nm tall, additional bump emerged at the line center, which was also indicated by a bright centerline in the according luminescent image. The line width slightly increased to approximately 100 μm.

At the highest frequency of 110 Hz, dot patterns with irregular dot sizes and distances between the dots were obtained instead of the desired line patterns as shown in Fig. 3(d). In addition, most of the deposited emitter material accumulated at the line center, forming a more than 100 nm tall peak. These observations suggested a loss of control over line dimensions and morphology at 110 Hz, possibly due to a disrupted ink flow during the jetting process.

### OLED device performance

**Fig. 4:**
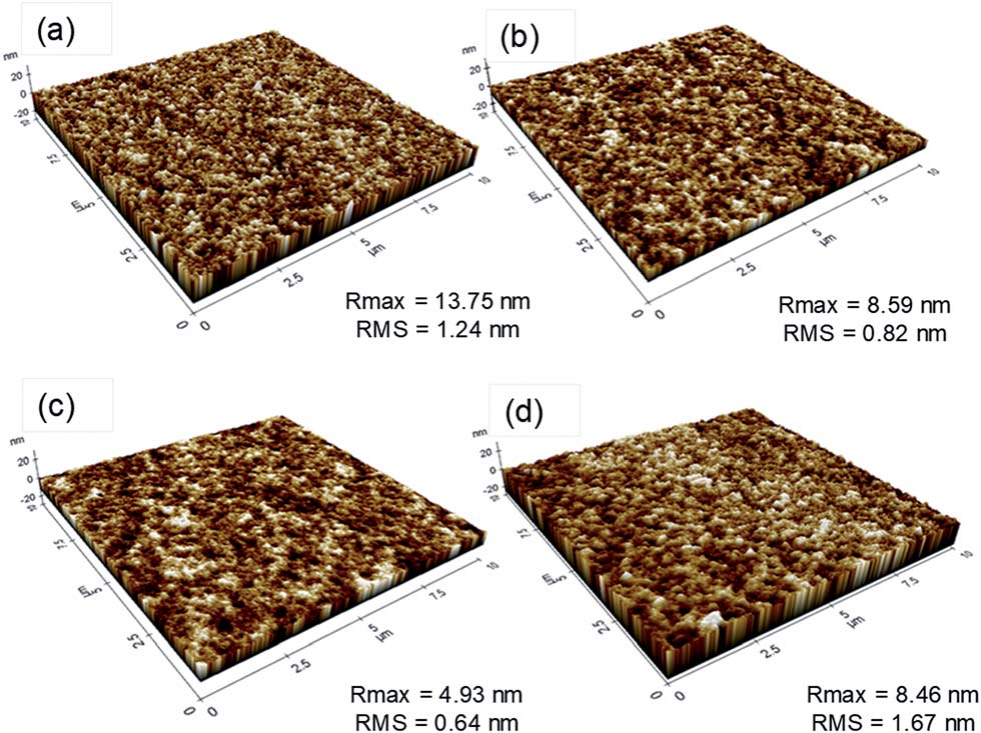
Surface morphology by AFM for (a) PEDOT:PSS (b) PVK/PEDOT:PSS (c) spin-coated SimCP2-FIrpic emitter on PVK/PEDOT:PSS (d) inkjet-printed SimCP2-FIrpic emitter on PVK/PEDOT:PSS.

The test devices shared the same architecture but differed in the technique used to deposit the emitting layer. First, we compared the surface roughness of each layer by successive film formation of spin-coated PEDOT:PSS, PVK (on PEDOT:PSS), and FIrpic-doped SimCP2 (on PVK/PEDOT:PSS). As illustrated in Fig. 4(b) and (c), the PVK and spin-coated SimCP2-FIrpic layers were smooth. The related *R*_max_ and RMS values, although not significantly low, suggested a non-aggregated condition thanks to good solubility of the functional materials in the solvents used.^22^ By contrast, the slightly increased RMS values obtained for the nanoparticle-based PEDOT:PSS surface (1.24 nm) and especially for the inkjet-printed SimCP2-FIrpic layer (1.67 nm) indicated a slightly aggregated condition, which can induce exciton quenching and hence reduced luminescent efficiency.^23^

**Fig. 5:**
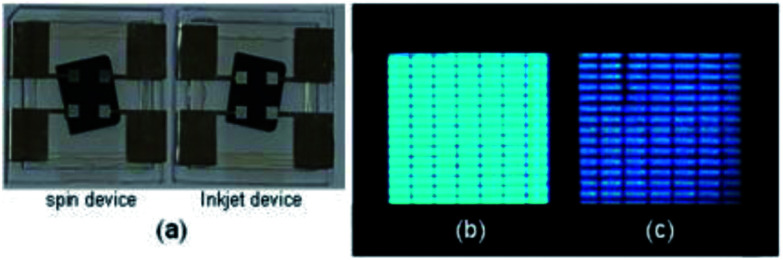
Device layout (a) with magnified image of light emitting pixels fabricated by spin casting (b) and inkjet printing (c).

Fig. 5 shows the layout (a) of the spin-coated and inkjet-printed OLED devices (25.4 mm × 25.4 mm) with 4 active light emitting cells of 2 mm × 2 mm, each having a 120 μm sub-pixel pitch (∼70 ppi). The pixel pattern accuracy corresponds to the resolution of a 65-inch RGB display with a 3840 × 2160 (4 K) resolution. Since the surface of the photo-patterned well was not additionally treated to prevent adherence of the host, the effective pixel areas seemed to be enlarged due to coverage with the emitter in case of the spin-coated EML (b). In contrast, the inkjet-printed device in Fig. 5(c) showed more accurately defined pixels across the photo-patterned well.

**Fig. 6:**
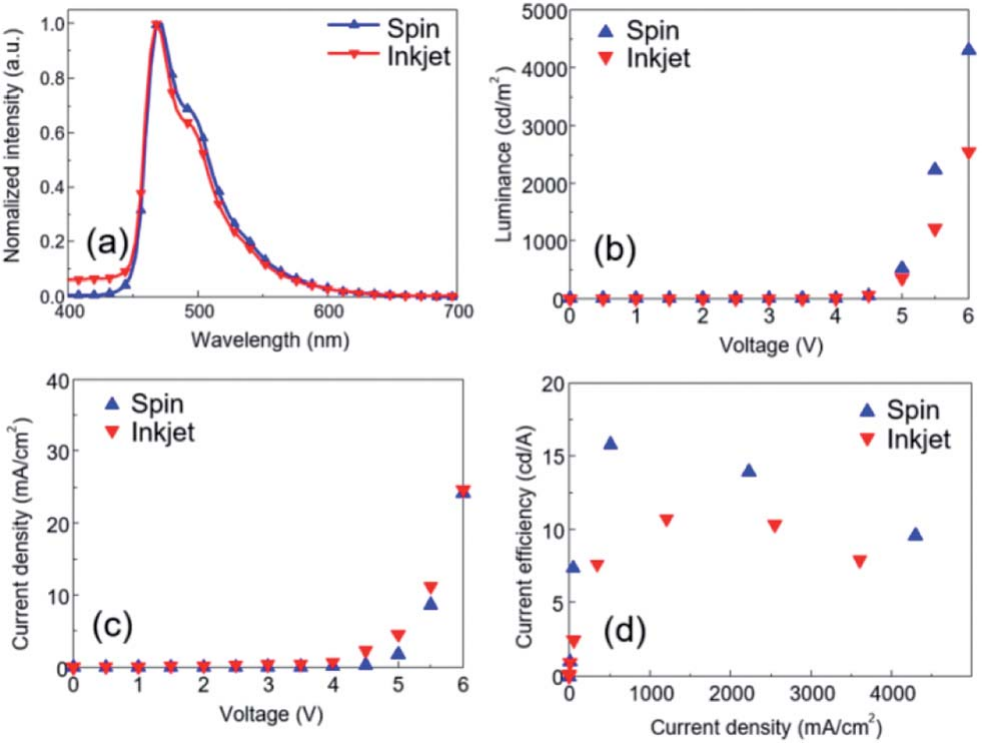
Comparison of device characteristics with spin-cast and inkjet-printed emitting layer: (a) electroluminescent spectra, (b) luminance *vs.* voltage, (c) current density *vs.* voltage, and (d) efficiency *vs.* luminance behavior.

The luminescence spectra obtained for both devices were almost identical, with a main peak at 468 nm and a pronounced secondary peak at 493 nm wavelength as shown in Fig. 6(a). The devices emitted sky-blue light, with color coordinates of (*X* 0.14, *Y* 0.25) and (*X* 0.14, *Y* 0.28) for the inkjet-printed and the spin-cast EML, respectively. The slight differences in color shade and spectra can be attributed to differences in the emitter thickness inside the patterned walls. During spin coating, the host molecules were subject to high radial forces causing the ink to form smooth and highly uniform layers inside the photo-patterned wells. This differs from the inkjet printing process where the evaporation dynamics determine the morphology of the emitting layer, resulting in generally thinner and less homogeneous layers inside the wells.

The devices differed in their brightness level and current density behavior as shown in Fig. 6(b) and (c). The device with the inkjet-printed EML produced a light output of around 2600 cd m^−2^ at 6.0 V, whereas 4300 cd m^−2^ were achieved with the spin-coated EML. This means that the device with the inkjet printed EML achieved around 60.5% of the reference device in terms of brightness. The differences between the two devices regarding current efficiency ( *J*) and power efficiency (PE) were slightly less pronounced (Table 2). A luminous efficiency of 10.73 cd A^−1^ and a power efficiency of 6.13 lm W^−1^ were obtained with the inkjet-printed EML, which amounted to 68% of what was possible with the spin-coated emitting layer.

**Table 2:** Device properties with spin-coated *vs.* inkjet-printed emitter

Devices	Properties		
	Efficiency (Cd A^−1^)_max_	Efficiency (lm W^−1^)_max_	CIE 1931 coordinates (*X*, *Y*)
Spin-coated EML	15.76	9.90	(0.14, 0.28)
Inkjet-printed EML	10.73	6.13	(0.14, 0.25)

The performance gap between the devices (Fig. 6(d)) can be related to differences in the height and surface roughness of the emitting layer. The spin-coated EML possessed a thickness of 25 nm and a very smooth (RMS = 0.64 nm) surface (Fig. 4(c)). The inferior performance of the inkjet-printed EML can be attributed to a less homogeneous surface morphology and an overall thicker layer. The thickness of the inkjet-printed EML varied from 22 nm (pixel center) to 30 nm (pixel edges). This was a result of the inkjet deposition process and the related evaporation dynamics, which did not only lower the light output and efficiency but also compromised the stability of the device in operation.

Our test device achieved a power efficiency of 9.90 lm W^−1^ with a spin-coated and 6.13 lm W^−1^ with an inkjet-printed EML. This was in a similar range compared to what was demonstrated for vacuum-processed mCP^3^ and only slightly lower than what was reported for vacuum-processed SimCP.^4,5^ However, the power efficiency of our devices amounted to only 25.3% and 40.9%, respectively, of the 24.2 lm W^−1^ stated by the Chen group for SimCP2,^6,7^ which can be attributed to the generally lower efficiency of solution-processed OLED devices and differences in the device architecture. Our device's current efficiency of 15.76 Cd A^−1^ with a spin-coated and 10.73 Cd A^−1^ with an inkjet-printed EML is equivalent to 50.7% and 34.5% of what was reported by Jou *et al.* for spin-coated SimCP2 at 1000 cd m^−2^.^8^ This demonstrates how the device efficiency further improves by increasing the dopant concentration and by working under nitrogen.

## Conclusions

Thin films of SimCP2 doped with blue-emitting FIrpic were successfully deposited by inkjet printing. Homogeneous patterns of the host material with controllable dimensions and smooth morphologies were obtainable when a viscous cosolvent (cyclohexanone) was added to the ink formulation at concentrations of 10–15 mol%. The best print results were observed at moderate print speed (3 mm s^−1^ at 70 Hz). A test device in [ITO/PEDOT:PSS /PVK/SimCP2:Flrpic/TSPO1/TPBi/LiF/Al] configuration was made in two versions. The device with the inkjet-printed emissive layer possessed excellent color fidelity (*X* 0.14; *Y* 0.25), a luminous efficiency of 10.73 cd A^−1^, and a power efficiency of 6.13 lm W^−1^. This was 68% of the luminous efficiency achieved from the same device with a spin-cast EML. Our results describe a path towards future applications of inkjet printing as a rapid and customizable micro-patterning technique for small molecular host materials in low-cost OLED devices. Future studies should investigate other combinations of small molecular host materials and solvents that can potentially enhance the homogeneity, efficiency and lifetime of blue-phosphorescent emitters deposited by inkjet printing.

## Conflicts of interest

There are no conflicts to declare.

## Supplementary Material

## References

[cit1] Wu M., Yeh S., Chen C., Murayama H., Tsuboi T., Li W., Chao I., Liu S., Wang J. (2007). Adv. Funct. Mater..

[cit2] Yook K., Lee J. (2012). Adv. Mater..

[cit3] Holmes R., Forrest S., Tung Y., Kwong R., Brown J., Garon S., Thompson M. (2003). Appl. Phys. Lett..

[cit4] Yeh S., Wu M., Chen C., Song Y., Chi Y., Ho M., Hsu S., Chen C. (2005). Adv. Mater..

[cit5] Tsuboi T., Murayama H., Yeh S., Wu M., Chen C. (2008). Opt. Mater..

[cit6] Jou J. , Wang W., Chen C., Chen S., Hsu M., Wang C., Wu M., Liu S., Shen S., Chen C. and Liu C., IDW’08 – Proc. 15th Int. Display Workshop, 2008, vol 2, pp. 1077–1078

[cit7] Tsuboi T., Liu S., Wu M., Chen C. (2009). Org. Electron..

[cit8] Jou J., Wang W., Chen S., Shyue J., Hsu M., Lin C., Shen S., Wang C., Liu C., Chen C., Wud M., Liu S. (2010). J. Mater. Chem..

[cit9] Alamán J., Alicante R., Peña J., Sánchez-Somolinos C. (2016). Materials.

[cit10] Jung S., Kim J., Kim H. (2012). Thin Solid Films.

[cit11] Xing R., Ye T., Ding Y., Ding Z., Ma D., Han Y. (2013). Chin. J. Chem..

[cit12] Yook K., Lee J. (2014). Adv. Mater..

[cit13] Liu H., Xu W., Tan W., Zhu X., Wang J., Peng J., Cao Y. (2016). J. Colloid Interface Sci..

[cit14] Deegan R., Bakajin O., Dupont T., Huber G., Nagel S., Witten T. (1997). Nature.

[cit15] Tekin E., de Gans B.-J., Schubert U. (2004). J. Mater. Chem..

[cit16] Hu H., Larson R. (2006). J. Phys. Chem. B.

[cit17] Soltman D., Subramanian V. (2008). Langmuir.

[cit18] Shen X., Ho C., Wong T. (2010). J. Phys. Chem. B.

[cit19] Friederich A., Binder J., Bauer W. (2013). J. Am. Ceram. Soc..

[cit20] de Gans B.-J., Schubert U. (2004). Langmuir.

[cit21] Chemical Engineering and Materials Research Information Center, Thermophysical properties data bank. https://www.cheric.org/research/kdb/hcprop/cmpsrch.php, 2017 (accessed 17.07.07)

[cit22] Kim Y., Wolf C., Cho H., Jeong S., Lee T. (2016). Adv. Mater..

[cit23] Cho Y., Yook K., Lee J. (2014). Adv. Mater..

